# Pregnancy following assisted reproductive technology in morbidly obese patients: assessment of feto-maternal outcomes

**DOI:** 10.1007/s10815-024-03065-1

**Published:** 2024-02-21

**Authors:** Bonnie B. Song, Rachel S. Mandelbaum, Zachary S. Anderson, Aaron D. Masjedi, Chelsey A. Harris, Caroline J. Violette, Joseph G. Ouzounian, Koji Matsuo, Richard J. Paulson

**Affiliations:** 1 Division of Gynecologic Oncology, Department of Obstetrics and Gynecology, University of Southern California, 2020 Zonal Avenue, IRD520, Los Angeles, CA 90033 USA; 2 Division of Reproductive Endocrinology and Infertility, Department of Obstetrics and Gynecology, University of Southern California, Los Angeles, CA USA; 3 Division of Maternal-Fetal Medicine, Department of Obstetrics and Gynecology, University of Southern California, Los Angeles, CA USA; 4 Norris Comprehensive Cancer Center, University of Southern California, Los Angeles, CA USA

**Keywords:** Pregnancy, Assisted reproductive technology, Morbid obesity, Intrauterine fetal demise, Severe maternal morbidity

## Abstract

**Purpose:**

To examine feto-maternal characteristics and outcomes of morbidly obese pregnant patients who conceived with assisted reproductive technology (ART).

**Methods:**

This cross-sectional study queried the Healthcare Cost and Utilization Project’s National Inpatient Sample. Study population was 48,365 patients with ART pregnancy from January 2012 to September 2015, including non-obesity (*n* = 45,125, 93.3%), class I–II obesity (*n* = 2445, 5.1%), and class III obesity (*n* = 795, 1.6%). Severe maternal morbidity at delivery per the Centers for Disease and Control Prevention definition was assessed with multivariable binary logistic regression model.

**Results:**

Patients in the class III obesity group were more likely to have a hypertensive disorder (adjusted-odds ratio (aOR) 3.03, 95% confidence interval (CI) 2.61–3.52), diabetes mellitus (aOR 3.08, 95%CI 2.64–3.60), large for gestational age neonate (aOR 3.57, 95%CI 2.77–4.60), and intrauterine fetal demise (aOR 2.03, 95%CI 1.05–3.94) compared to those in the non-obesity group. Increased risks of hypertensive disease (aOR 1.35, 95%CI 1.14–1.60) and diabetes mellitus (aOR 1.39, 95%CI 1.17–1.66) in the class III obesity group remained robust even compared to the class I–II obesity group. After controlling for priori selected clinical, pregnancy, and delivery factors, patients with class III obesity were 70% more likely to have severe maternal morbidity at delivery compared to non-obese patients (8.2% vs 4.4%, aOR 1.70, 95%CI 1.30–2.22) whereas those with class I–II obesity were not (4.1% vs 4.4%, aOR 0.87, 95%CI 0.70–1.08).

**Conclusions:**

The results of this national-level analysis in the United States suggested that morbidly obese pregnant patients conceived with ART have increased risks of adverse fetal and maternal outcomes.

**Supplementary Information:**

The online version contains supplementary material available at 10.1007/s10815-024-03065-1.

## Introduction

Obesity is a common comorbidity with increasing incidence in the United States. Current trends project that nearly half of U.S. adults will be obese by 2030, with > 20% being severely obese (body mass index (BMI) of ≥ 35 kg/m^2^) [1]. Severe obesity is projected to be the most common subcategory among adults in 10 U.S. states by 2030 [1]. Severe obesity further increases the risk of obesity-related complications such as cardiovascular disease and diabetes [2, 3].

In pregnancy, obesity is associated with an increased risk for adverse maternal and fetal outcomes, including miscarriage, preeclampsia, gestational diabetes, and cesarean delivery [4, 5]. While many of these risks have been well described in the literature [6–9], further subgroup analysis in patients with severe obesity has been relatively limited.

Obese patients are more likely to experience infertility due to menstrual dysfunction and anovulation [10, 11]. This may be related to altered secretion of pulsatile gonadotropin hormone-releasing hormone and reduced gonadotropin receptiveness due to increased peripheral aromatization of androgens to estrogens, as well as insulin resistance and hyperinsulinemia leading to hyperandrogenism [12–14]. Adipose tissue also secretes adipokines, which are linked to subfertility [15]. Rates of infertility can be threefold higher in obese patients, and obese patients need longer time to achieve pregnancy [16–18].

Given the association of obesity and infertility, these patients are more likely to require infertility services. Studies evaluating fertility outcomes in obese patients with assisted reproductive technology (ART) are mixed. Some suggest obese patients require higher doses of medications to induce ovulation, while experiencing decreased odds of ovulation in response to clomiphene citrate [19–21]. Additionally, some suggest that obesity is associated with decreases in oocytes retrieved, decreased endometrial receptivity, lower egg quality, and lower rates of live birth [22, 23]. Meanwhile, other meta-analyses and retrospective cohort studies found that obesity was not associated with poorer in vitro fertilization (IVF) outcomes such as decreased implantation rates, miscarriage rates, or live birth rates [24–26].

Despite the variety of studies evaluating the effects of general obesity on fertility and IVF outcomes, there is limited data on feto-maternal outcomes among pregnancies conceived with ART in patients with severe obesity (also known as morbid obesity). The objective of this study was to examine feto-maternal characteristics and outcomes of morbidly obese patients who conceived with ART.

## Methods

### Data source

This cross-sectional study queried the Nationwide (National) Inpatient Sample (NIS) that was developed for the Healthcare Cost and Utilization Project (HCUP) that is supported by the Agency for Healthcare Research and Quality (AHRQ) [27], one of the twelve federal agencies within the United States Department of Health and Human Service (HHS).

The NIS program is a population-based all-payer database for inpatient records selecting randomly 20% in each year capturing all the admission cases, and the weighted data for national estimates represents more than 90% of the U.S. population. We chose NIS as this represents the largest admission data source in this country and is the important resource to assess maternal delivery outcomes. The University of Southern California Institutional Review Board exempted this study due to the use of publicly available deidentified data.

### Eligibility criteria

The study population was hospital deliveries among pregnant patients following ART between January 2012 and September 2015. The identification of ART was based on the World Health Organization’s International Classification of Disease 9th revision (ICD-9) codes of V23.85 per prior study (Supplemental Table S1). The ICD-9 code does not provide details of ART such as IVF, artificial insemination, or intracytoplasmic sperm injection.

The study starting point was chosen as the NIS data capturing mechanism was redesigned in 2012 to include data from all hospitals instead of a sample of participating hospitals to improve national estimates. The study end point of September 2015 was chosen given the transition of ICD-9 codes in the NIS program and to ensure consistency in coding across the study period. More recent year cases were not examined in this study due to possible undercapture of ART pregnancy in the program. Vaginal and cesarean deliveries were identified based on the ICD-9 codes and Disease-Related Group codes (Supplemental Table S1). Patients with missing age were excluded from the analysis.

### Exposure assignment

Patients who met the study inclusion criteria were grouped based on body habitus into the following three categories: non-obesity, class I–II obesity, and class III obesity. Obesity categories in this study followed the Centers for Disease Control and Prevention (CDC) classification: BMI of 30–34.9 kg/m^2^ for class I obesity, 35–39.9 kg/m^2^ for class II obesity, and ≥ 40 kg/m^2^ for class III obesity, respectively (Supplemental Table S1) [28]. This subdivision of class I–II and III obesity followed prior investigations for morbid obesity. Patients with absence of these ICD-9 codes for obesity were categorized in the non-obesity group.

### Outcome measures

The main outcome measures were severe maternal morbidity (SMM) at delivery related to body habitus (non-obesity, class I–II obesity, and class III obesity). This study followed the CDC definition for identifying SMM, and a total of 21 indicators for SMM were evaluated: acute myocardial infarction, aneurysm, acute renal failure, adult respiratory distress syndrome, amniotic fluid embolism, cardiac arrest/ventricular fibrillation, cardiac rhythm conversion, disseminated intravascular coagulation, eclampsia, heart failure/arrest during surgery or procedure, puerperal cerebrovascular disorders, pulmonary edema/acute heart failure, severe anesthesia complications, sepsis, shock, sickle cell disease with crisis, air and thrombotic embolism, blood products transfusion, hysterectomy, temporary tracheostomy, and ventilation [29].

Additionally, hemorrhage, length of hospital admission, and total charge for the index admission for hospital delivery were examined. The total charge was corrected for the 2023 value based on the medical inflation rate [30].

### Study covariates

The study covariates examined were preselected in a view of relevance to the exposure and outcomes. These included 13 clinical factors and 15 pregnancy and delivery factors (a total of 28 factors). The identification of these study covariates was based on the program-defined information and aggregation per the ICD-9 codes (Supplemental Table S1). Cases with unknown data were grouped as one category in each variable.

Clinical factors included patient age (< 35 or ≥ 35 years), year (2012, 2013, 2014, and 2015), race and ethnicity (White, Black, Hispanic, Asian, and other) determined by the National Inpatient Sample program, primary expected payer (private including HMO, Medicaid, and other), census-level median household income (every quartile), medical comorbidity (pregestational hypertension and pregestational diabetes mellitus), substance factor (tobacco use), and uterine factor (prior cesarean scar and uterine myoma). Hospital parameters included hospital relative bed capacity (small, mid, or large), hospital location and teaching status (rural, urban non-teaching, or urban teaching), and hospital region in the United States (Northeast, Midwest, South, and West). Race and ethnicity were examined as this factor is associated with pregnancy and delivery characteristics and outcomes.

Pregnancy factors included the following: (i) fetal factors (multifetal gestation, intrauterine fetal growth restriction, intrauterine fetal demise, large for gestational age, and breech presentation), (ii) placental factor (placenta previa, placenta abruption, and placenta accreta spectrum), (iii) membranous factors (preterm premature rupture of membrane and chorioamnionitis), (iv) maternal factors (gestational hypertension, preeclampsia, and gestational diabetes mellitus), and (v) delivery factors (preterm birth and delivery route (vaginal or cesarean)). These study covariates were identified and aggregated according to the ICD-9 code schema (Supplemental Table S1).

### Statistical analysis

The first step of analysis was to compute the proportional distribution of the clinical and pregnancy characteristics per the exposure groups (non-obesity, class I–II obesity, and class III obesity), expressed with percentage per group. Statistical difference among the three exposure groups was then assessed with the Pearson chi-square test.

The second step of analysis was to identify the independent clinical and pregnancy characteristics associated with the maternal body habitus. A multinomial regression model was fitted for this step analysis. All the baseline clinical and pregnancy covariates exhibiting a *P*-value of less than 0.05 in univariable analysis were considered in the initial model selections. Conditional backward selection was then used for the final model selection in this study. The effect size for class I–II obesity or class III obesity compared to non-obesity was estimated as adjusted-odds ratio (aOR) with a corresponding 95% confidence interval (CI).

The third step of analysis was to assess the exposure-outcome association. SMM, hemorrhage, prolonged admission defined as 7 days or longer hospital stay for delivery, and corrected total charge were examined for the class I–II obesity group and the class III obesity group comparing to the non-obesity group. A binary logistic regression model with parsimonious adjustment was fitted to assess the exposure-outcome relationship. The adjusting factors were predefined in relevance of the exposure and maternal outcome, including clinical and pregnancy factors that are (i) different in the exposure groups and/or (ii) historically known for SMM. Pregnancy factors were also considered to reflect the chronology that may be mediated by difference in the baseline clinical factors. These included patient age, race and ethnicity, prior cesarean delivery, hypertensive disorder, diabetes mellitus, placenta previa, placenta abruption, placenta accreta spectrum, multifetal gestation, delivery type, and hospital bed capacity. The magnitude of significance was expressed with aOR and a corresponding 95%CI.

Various sensitivity analyses were conducted to assess the robustness of study findings. First, the class III obesity group was compared to the class I–II obesity group. Second, each individual SMM indicator was assessed. Third, interaction-term analysis was performed to assess the significance of body habitus on hemorrhage and blood transfusion. This evaluation was based on the post hoc observation of increased risks of both outcomes in class III obese patients. Fourth, the exposure-outcome association was assessed by excluding cases with unknown information.

The weights for national estimates provided by the National Inpatient Sample were used for analysis. Statistical interpretation followed a two-tailed hypothesis, and a *P*-value of less than 0.05 was considered statistically significant. IBM SPSS Statistics (version 28.0, Armonk, NY, USA) was used for all analysis. The current study followed the STROBE reporting guidelines to summarize the performance of the results.

## Results

### Study cohort

During the study period, 48,365 hospital deliveries were recorded for pregnant patients who conceived with ART. This study cohort comprised of 45,125 (93.3%) patients with non-obesity, 2445 (5.1%) patients with class I–II obesity, and 795 (1.6%) patients with class III obesity.

### Clinical, pregnancy, and delivery characteristics

In univariable analyses for clinical characteristics (Table 1), year of delivery, race and ethnicity, census-level household income, tobacco use, pregestational hypertension, pregestational diabetes, uterine myoma, hospital location and teaching status, and hospital region were statistically significantly associated with the exposure (all, *P* < 0.05).

**Table 1:** Clinical demographics

Characteristic	Non-obesity	Class I–II obesity	Class III obesity	*P*-value
No	45,125 (100)	2445 (100)	795 (100)	
Age (y)	35 (32–39)	35 (32–39)	36 (33–40)	0.465
< 35	19,735 (43.7)	1090 (44.6)	335 (42.1)	
≥ 35	25,390 (56.3)	1355 (55.4)	460 (57.9)	
Year				< 0.001
2012	9150 (20.3)	475 (19.4)	165 (20.8)	
2013	11,215 (24.9)	480 (19.6)	175 (22.0)	
2014	13,795 (30.6)	775 (31.7)	240 (30.2)	
2015	10,965 (24.3)	715 (29.2)	215 (27.0)	
Race/ethnicity				< 0.001
White	30,040 (66.6)	1595 (65.2)	520 (65.4)	
Black	2275 (5.0)	195 (8.0)	80 (10.1)	
Hispanic	2655 (5.9)	225 (9.2)	45 (5.7)	
Asian	5445 (12.1)	165 (6.7)	40 (5.0)	
Other	2400 (5.3)	75 (3.1)	60 (7.5)	
Unknown	2310 (5.1)	190 (7.8)	50 (6.3)	
Primary payer				0.091
Private	41,665 (92.3)	2250 (92.0)	735 (92.5)	
Medicaid	1715 (3.8)	110 (4.5)	40 (5.0)	
Other	1735 (3.8)	85 (3.5)	20 (2.5)	
Unknown	**	0	0	
Household income				< 0.001
QT1 (lowest)	3215 (7.1)	160 (6.5)	85 (10.7)	
QT2	5675 (12.6)	385 (15.7)	150 (18.9)	
QT3	10,665 (23.6)	720 (29.4)	225 (28.3)	
QT4 (highest)	25,125 (55.7)	1155 (47.2)	325 (40.9)	
Unknown	445 (1.0)	25 (1.0)	**	
Tobacco use				0.004
No	44,940 (99.6)	2445 (100)	790 (99.4)	
Yes	185 (0.4)	0	**	
Pregestational hypertension				< 0.001
No	43,675 (96.8)	2150 (87.9)	620 (78.0)	
Yes	1450 (3.2)	295 (12.1)	175 (22.0)	
Pregestational diabetes				< 0.001
No	44,710 (99.1)	2365 (96.7)	720 (90.6)	
Yes	415 (0.9)	80 (3.3)	75 (9.4)	
Prior cesarean delivery				0.608
No	39,070 (86.6)	2100 (85.9)	690 (86.8)	
Yes	6055 (13.4)	345 (14.1)	105 (13.2)	
Uterine myoma				< 0.001
No	42,425 (94.0)	2225 (91.0)	735 (92.5)	
Yes	2700 (6.0)	220 (9.0)	60 (7.5)	
Hospital bed capacity				0.127
Small	5555 (12.3)	285 (11.7)	80 (10.1)	
Mid	12,970 (28.7)	675 (27.6)	225 (28.3)	
Large	26,600 (58.9)	1485 (60.7)	490 (61.6)	
Hospital teaching				< 0.001
Rural	1010 (2.2)	45 (1.8)	15 (1.9)	
Urban non-teaching	9970 (22.1)	425 (17.4)	165 (20.8)	
Urban teaching	34,145 (75.7)	1975 (80.8)	615 (77.4)	
Hospital region				< 0.001
Northeast	15,325 (34.0)	710 (29.0)	230 (28.9)	
Midwest	7165 (15.9)	580 (23.7)	140 (17.6)	
South	10,690 (23.7)	565 (23.1)	215 (27.0)	
West	11,945 (26.5)	590 (24.1)	210 (26.4)	

Median (IQR) or percentage per column is shown. Pearson chi-square test for *P*-values. **Small number suppressed per the HCUP guidelines. Abbreviation: *QT* quartile

The results of univariable analysis for pregnancy and delivery characteristics per the exposure are displayed in Table 2. (i) Fetal factors including multifetal gestation, intrauterine fetal demise, large for gestational age, and breech presentation; (ii) placental factors with placenta previa and placental abruption; (iii) membranous factor with chorioamnionitis; (iv) maternal factors with gestational hypertension, preeclampsia, and gestational diabetes mellitus; and *(v)* delivery factors with preterm birth and delivery route reached statistical threshold (all, *P* < 0.05).

**Table 2:** Pregnancy and delivery characteristics

Characteristic	Non-obesity	Class I–II obesity	Class III obesity	*P*-value
No	45,125 (100)	2445 (100)	795 (100)	
Multifetal gestation				0.004
No	32,200 (71.4)	1735 (71.0)	525 (66.0)	
Yes	12,925 (28.6)	710 (29.0)	270 (34.0)	
Fetal growth restriction				0.863
No	42,235 (93.6)	2295 (93.9)	745 (93.7)	
Yes	2890 (6.4)	150 (6.1)	50 (6.3)	
Fetal demise				0.002
No	44,900 (99.5)	2425 (99.2)	785 (98.7)	
Yes	225 (0.5)	20 (0.8)	**	
Breech presentation				< 0.001
No	39,630 (87.8)	2130 (87.1)	610 (76.7)	
Yes	5495 (12.2)	315 (12.9)	185 (23.3)	
Large for gestational age				< 0.001
No	43,805 (97.1)	2230 (91.2)	720 (90.6)	
Yes	1320 (2.9)	215 (8.8)	75 (9.4)	
Placenta previa				0.004
No	43,910 (97.3)	2405 (98.4)	770 (96.9)	
Yes	1215 (2.7)	40 (1.6)	25 (3.1)	
Placental abruption				0.012
No	44,155 (97.9)	2390 (97.8)	790 (99.4)	
Yes	970 (2.1)	55 (2.2)	**	
Placenta accreta spectrum				0.089
No	44,955 (99.6)	2440 (99.8)	795 (100)	
Yes	170 (0.4)	**	0	
PPROM				0.071
No	40,945 (90.7)	2225 (91.0)	740 (93.1)	
Yes	4180 (9.3)	220 (9.0)	55 (6.9)	
Chorioamnionitis				0.005
No	43,350 (96.1)	2320 (94.9)	755 (95.0)	
Yes	1775 (3.9)	125 (5.1)	40 (5.0)	
Gestational hypertension				< 0.001
No	42,690 (94.6)	2235 (91.4)	735 (92.5)	
Yes	2435 (5.4)	210 (8.6)	60 (7.5)	
Preeclampsia				< 0.001
No	40,605 (90.0)	1975 (80.8)	595 (74.8)	
Yes	4520 (10.0)	470 (19.2)	200 (25.2)	
Gestational diabetes				< 0.001
No	39,615 (87.8)	1880 (76.9)	605 (76.1)	
Yes	5510 (12.2)	565 (23.1)	190 (23.9)	
Preterm birth				0.033
No	38,065 (84.4)	2015 (82.4)	675 (84.9)	
Yes	7060 (15.6)	430 (17.6)	120 (15.1)	
Delivery route				< 0.001
Vaginal	19,160 (42.5)	745 (30.5)	240 (30.2)	
Cesarean	25,965 (57.5)	1700 (69.5)	555 (69.8)	

Percentage per column is shown. Pearson chi-square test for *P*-values. **Small number suppressed per the HCUP guidelines. Abbreviation: *PPROM* preterm premature rupture of membrane

### Independent characteristics

The results of multivariable analyses are shown in Table 3. Patients in the class III obesity group were more likely to have hypertensive disorders (aOR 3.03, 95%CI 2.61–3.52) and diabetes mellitus (aOR 3.08, 95%CI 2.64–3.60) compared to those in the non-obesity group that the effect size exceeded twofold among all the independent characteristics.

**Table 3:** Multivariable analysis

Variables*	Class I–II obesity (vs non-obesity)		Class III obesity (vs non-obesity)	
No	aOR (95%CI)	*P*-value	aOR (95%CI)	*P*-value
Year				
2012	1.00 (reference)		1.00 (reference)	
2013	0.86 (0.75–0.98)	0.023	0.95 (0.76–1.18)	0.614
2014	1.06 (0.94–1.19)	0.361	0.98 (0.80–1.21)	0.863
2015	1.22 (1.08–1.38)	0.002	1.08 (0.87–1.33)	0.493
Race/ethnicity				
White	1.00 (reference)		1.00 (reference)	
Black	1.43 (1.21–1.68)	< 0.001	1.65 (1.28–2.12)	< 0.001
Hispanic	1.65 (1.42–1.91)	< 0.001	0.90 (0.66–1.23)	0.510
Asian	0.55 (0.46–0.65)	< 0.001	0.38 (0.27–0.53)	< 0.001
Other	0.61 (0.48–0.77)	< 0.001	1.39 (1.05–1.84)	0.020
Unknown	1.32 (1.12–1.56)	0.001	1.17 (0.86–1.59)	0.326
Census-level household income				
QT1 (lowest)	1.00 (reference)		1.00 (reference)	
QT2	1.48 (1.22–1.80)	< 0.001	1.13 (0.86–1.49)	0.395
QT3	1.50 (1.26–1.80)	< 0.001	0.92 (0.71–1.19)	0.527
QT4 (highest)	1.15 (0.96–1.37)	0.127	0.62 (0.48–0.80)	< 0.001
Unknown	1.39 (0.89–2.16)	0.148	1.02 (0.52–2.01)	0.951
Hypertensive disorder				
No	1.00 (reference)		1.00 (reference)	
Yes	2.25 (2.05–2.46)	< 0.001	3.03 (2.61–3.52)	< 0.001
Diabetes mellitus				
No	1.00 (reference)		1.00 (reference)	
Yes	2.21 (2.01–2.44)	< 0.001	3.08 (2.64–3.60)	< 0.001
Uterine myoma				
No	1.00 (reference)		1.00 (reference)	
Yes	1.36 (1.17–1.58)	< 0.001	1.03 (0.78–1.35)	0.856
Hospital teaching				
Rural	0.76 (0.68–0.85)	< 0.001	0.69 (0.41–1.18)	0.173
Urban non-teaching	0.68 (0.50–0.93)	0.015	0.94 (0.79–1.13)	0.524
Urban teaching	1.00 (reference)		1.00 (reference)	
Hospital region				
Northeast	0.96 (0.85–1.08)	0.469	0.85 (0.70–1.04)	0.111
Midwest	1.48 (1.31–1.68)	< 0.001	0.93 (0.74–1.16)	0.497
South	0.96 (0.85–1.09)	0.567	0.95 (0.78–1.17)	0.645
West	1.00 (reference)		1.00 (reference)	
Fetal demise				
No	1.00 (reference)		1.00 (reference)	
Yes	1.62 (1.01–2.58)	0.045	2.03 (1.05–3.94)	0.036
Breech presentation				
No	1.00 (reference)		1.00 (reference)	
Yes	0.93 (0.82–1.05)	0.241	2.02 (1.69–2.41)	< 0.001
Large for gestational age				
No	1.00 (reference)		1.00 (reference)	
Yes	3.23 (2.76–3.78)	< 0.001	3.57 (2.77–4.60)	< 0.001
Placenta previa				
No	1.00 (reference)		1.00 (reference)	
Yes	0.52 (0.37–0.72)	< 0.001	1.09 (0.72–1.65)	0.696
Placental abruption				
No	1.00 (reference)		1.00 (reference)	
Yes	1.07 (0.81–1.42)	0.621	0.31 (0.13–0.74)	0.009
Chorioamnionitis				
No	1.00 (reference)		1.00 (reference)	
Yes	1.41 (1.17–1.71)	< 0.001	1.55 (1.11–2.16)	0.009
Preterm birth				
No	1.00 (reference)		1.00 (reference)	
Yes	1.06 (0.95–1.19)	0.304	0.75 (0.61–0.92)	0.005
Delivery type				
Vaginal	1.00 (reference)		1.00 (reference)	
Cesarean	1.41 (1.29–1.55)	< 0.001	1.16 (0.99–1.37)	0.071

A multinomial regression model for analysis. All the listed covariates were entered in the model. Non-obesity group served as the reference group. Results are similar when unknown cases were excluded (6.2%). *Clinical and pregnancy characteristics. Abbreviations: *aOR* adjusted odds ratio, *CI* confidence interval, *QT* quartile

These increased risks of hypertensive disease (aOR 1.35, 95%CI 1.14–1.60) and diabetes mellitus (aOR 1.39, 95%CI 1.17–1.66) in the class III obesity group remained robust even compared to the class I–II obesity group.

Moreover, class III obesity pregnancy was independently associated with increased risks of large for gestational age (aOR 3.57, 95%CI 2.77–4.60), breech presentation (aOR 2.02, 95%CI 1.69–2.41), and intrauterine fetal demise (aOR 2.03, 95%CI 1.05–3.94) compared to non-obesity and the odds were greater than twofold for all these characteristics (Table 3).

Patients in the class I–II obesity group were also more likely to have hypertensive disorder, diabetes mellitus, large for gestational age, and intrauterine fetal demise compared to those in the non-obesity group, but the odds were smaller compared to the class III obesity group (Table 3).

### Maternal morbidity at delivery

Outcome data are shown in Table 4. After controlling for priori selected clinical, pregnancy, and delivery factors, patients with class III obesity were 70% more likely to have SMM at delivery compared to non-obese patients (8.2% vs 4.4%, aOR 1.70, 95%CI 1.30–2.22). Patients in the class I–II obesity group have similar SMM compared to those in the non-obesity group (4.1% vs 4.4%, aOR 0.87, 95%CI 0.70–1.08).

**Table 4:** Maternal morbidity at delivery

		Unadjusted		Adjusted*	
Outcome	Rate (%)	OR (95%CI)	*P*-value	aOR (95%CI)	*P*-value
SMM (any)					
Non-obesity	4.4	1.00 (reference)		1.00 (reference)	
Class I–II obesity	4.1	0.93 (0.76–1.15)	0.515	0.87 (0.70–1.08)	0.207
Class III obesity	8.2	1.95 (1.51–2.52)	< 0.001	1.70 (1.30–2.22)	< 0.001
Hemorrhage (any)					
Non-obesity	10.9	1.00 (reference)		1.00 (reference)	
Class I–II obesity	12.9	1.21 (1.07–1.37)	0.002	1.19 (1.05–1.35)	0.006
Class III obesity	15.1	1.46 (1.20–1.77)	< 0.001	1.36 (1.11–1.66)	0.003
Blood transfusion (any)					
Non-obesity	3.2	1.00 (reference)		1.00 (reference)	
Class I–II obesity	2.7	0.84 (0.65–1.07)	0.159	0.79 (0.61–1.03)	0.076
Class III obesity	6.3	2.05 (1.53–2.74)	< 0.001	1.80 (1.33–2.43)	< 0.001
Hemorrhage (no transfusion)					
Non-obesity	8.6	1.00 (reference)		1.00 (reference)	
Class I–II obesity	10.6	1.26 (1.10–1.44)	< 0.001	1.19 (1.04–1.36)	0.013
Class III obesity	11.3	1.41 (1.13–1.76)	0.003	1.28 (1.02–1.61)	0.030
Transfusion (no hemorrhage)					
Non-obesity	0.9	1.00 (reference)		1.00 (reference)	
Class I–II obesity	**	0.47 (0.25–0.88)	0.018	0.41 (0.22–0.78)	0.006
Class III obesity	2.5	3.04 (1.93–4.79)	< 0.001	2.56 (1.59–4.12)	< 0.001
Hemorrhage + transfusion					
Non-obesity	2.3	1.00 (reference)		1.00 (reference)	
Class I–II obesity	2.2	1.00 (0.76–1.32)	0.984	0.94 (0.71–1.25)	0.681
Class III obesity	3.8	1.77 (1.22–2.57)	0.003	1.47 (1.01–2.15)	0.046
Length of stay ≥ 7 days					
Non-obesity	6.6	1.00 (reference)		1.00 (reference)	
Class I–II obesity	12.3	1.97 (1.74–2.24)	< 0.001	1.63 (1.42–1.87)	< 0.001
Class III obesity	15.1	2.51 (2.06–3.05)	< 0.001	1.76 (1.42–2.19)	< 0.001

*The exposure-outcome association was adjusted for priori selected clinical, pregnancy, and delivery characteristics (patient age, race and ethnicity, prior cesarean delivery, hypertensive disorder, diabetes mellitus, placenta previa, placenta abruption, placenta accreta spectrum, multifetal gestation, delivery type, and hospital bed capacity). The results were similar by excluding cases with unknown information in study covariate (6.2%). Abbreviation: *SMM* severe maternal morbidity

**Small number suppressed per the HCUP guidelines

Risks of hemorrhage (15.1% vs 10.9%, aOR 1.36, 95%CI 1.11–1.66) and blood transfusion (6.3% vs 3.2%, aOR 1.80, 95%CI 1.33–2.43) were both increased in the class III obesity group compared to the non-obese group.

In an interaction-term analysis examining hemorrhage and blood transfusion (Table 4), risks of hemorrhage that did not require blood transfusion (aOR 1.28, 95%CI 1.02–1.61), blood transfusion for condition without hemorrhage (aOR 2.56, 95%CI 1.59–4.12), and blood transfusion for hemorrhage (aOR 1.47, 95%CI 1.01–2.15) were all increased in the class III obese group compared to non-obese group. This association was not observed in the class I–II obesity group.

The median total charge for the hospital admission for delivery in the class III obesity group was higher compared to other two groups: $29,251 for class III obesity; $26,048 for class I–II obesity; and $25,193 for the non-obesity group, respectively (*P* < 0.001). Both obesity groups demonstrated increased likelihood of prolonged hospital stay for delivery compared to those in the non-obesity group (both, *P* < 0.001; Table 4).

## Discussion

### Principal findings

This analysis found that patients with morbid obesity conceiving through ART may be at greater risk for multiple adverse obstetric outcomes and experience higher risks of maternal morbidity at delivery. This evaluation is critical at a time when the populations of morbid obesity and infertility are continuing to increase.

### Insights for results

#### Maternal characteristics

Other studies have found that patients with general obesity and ART pregnancies were more likely to experience gestational diabetes and hypertensive disorders [31–33]. With national-level data, our study reinforces this association for class III obesity.

There are several possible explanations. Firstly, ART use itself is associated with a higher risk for hypertensive disorders of pregnancy and gestational diabetes [33, 34]. Patients who require ART to conceive differ from those who do not in that they are older and more often nulliparous, both of which are risk factors for preeclampsia. They may be anovulatory due to polycystic ovary syndrome, which may be further associated with metabolic syndrome. Additionally, ART treatment may predispose patients to gestational hypertensive disorders via impaired endometrial receptivity resulting from repeated cycles of ovarian stimulation [35, 36]. The abnormal trophoblastic invasion in a poorly receptive maternal endometrium may then develop preeclampsia. As obesity is also associated with decreased endometrial receptivity [22, 23], severe maternal obesity may compound this risk.

Furthermore, patients with class II–III obesity tend to require higher doses of ovarian stimulation and gonadotropins to achieve pregnancy [19, 21], which may contribute to impaired placentation and development of preeclampsia. Lastly, the association of obesity and infertility with insulin resistance [13, 14] may also contribute to the predisposition of severely obese patients with ART pregnancies to developing gestational diabetes.

#### Pregnancy characteristics

Some studies have found that maternal obesity is associated with preterm delivery in IVF pregnancies [37]. Our study population, while not limited specifically to patients undergoing IVF but all forms of ART, notes increased rates of preterm birth among patients with class III obesity, but not among class I–II obesity. Since patients with class III obesity who conceived through ART are more likely to experience pregnancy complications such as preeclampsia and gestational diabetes as described above, they are more likely to have indications for preterm delivery. Additionally, larger maternal habitus may increase adipokines and secretion of proinflammatory cytokines, which may be implicated in preterm birth [15, 38].

We also note higher rates of intrauterine fetal demise in class II and class III obesity, which may help to elucidate the mixed data on this relationship. Some studies suggest that general obesity is associated with increased miscarriage rates and decreased live birth rates in IVF pregnancies [22, 39, 40], while other studies did not find a significant association [25, 26, 37]. As obesity and ART use are associated with abnormal trophoblastic invasion and increased risk for placental insufficiency [41, 42], this may explain the increase in intrauterine fetal demise noted among obese patients with ART pregnancies, which becomes even more prominent in class III obesity. Furthermore, some studies have hypothesized that a higher rate of pregnancy loss may be related to impaired endometrial receptivity and embryo quality in overweight and obese patients [14, 39].

The increased incidence of large for gestational age among obese patients with ART pregnancies was also noted in other retrospective cohort studies [31, 43, 44]. While IVF pregnancy itself does not seem to be associated with incidence of large for gestational age, there appears to be an increase in incidence of large for gestational age neonates among obese mothers who conceive through IVF [44]. Factors associated with obesity such as gestational and pregestational diabetes are likely contributory. While human data is limited, embryo culture media appear to affect fetal growth rate and birthweight of neonates [45, 46]. It is unclear if morbid obesity further compounds the risk of having a large for gestational age neonate when the pregnancy is conceived through ART.

#### Maternal morbidity

Our study provides a unique analysis of SMM and risks of hemorrhage and transfusion among morbidly obese patients with ART pregnancies. Recent studies suggest that IVF may be associated with placental abnormalities and postpartum hemorrhage [47, 48]. Additionally, a 2018 retrospective study found that IVF is associated with SMM compared to non-IVF pregnancies, but the SMM risk was not higher among obese individuals than non-obese individuals [49]. Our data in comparison includes a larger population of patients receiving ART and patients who experienced SMM, in addition to obesity subgroup analysis. We also did not find significant differences in SMM among class I–II obesity, but we did in class III obesity.

This analysis is one of the few investigations to analyze the association of morbid obesity with hemorrhage and blood transfusion in ART pregnancies. Notably, the odds of blood transfusion are increased even in the absence of hemorrhage. Reasons for this association are unclear; however, the higher incidence of transfusion in the morbidly obese population could raise the possibility of decreased compensatory reserve in response to blood loss during delivery. Whether the increased risk for hemorrhage and transfusion is due to ART treatments themselves or maternal factors associated with infertility and/or obesity remains unclear.

Maternal factors such as uterine myoma and adenomyosis are associated with both obesity and infertility and are known risk factors for hemorrhage [50–52]. We did not notice a significantly increased risk of placental abnormalities such as placenta previa or placental abruption in our study population, although some studies have noted such an association with IVF pregnancies and considered this as a contributor to postpartum hemorrhage [41, 48, 51].

### Strengths and limitations

While many of the results were in part reported in previous investigations, nationwide data capturing schema and enhanced study covariates strengthened the interpretation of study findings, and data on subclassification for obesity in this study adds important information in the literature.

There are several limitations in this study. Most importantly, there are unmeasured confounders that may possibly alter the observed exposure-outcome association in this retrospective study. These include details of ART such as type (IVF, intrauterine insemination, and intracytoplasmic sperm injection) and indication (maternal or paternal factor). Accuracy of ART pregnancy was also not assessable due to lack of actual medical record review. Lack of information on BMI in the ICD-9 coding schema is another limitation, and there is a possibility of undercapture in obese groups. Severe maternal outcomes were only assessed by the administrative codes for CDC’s instrumental variables that may not completely capture all the severe outcomes. Definition and cause of hemorrhage as well as extent of blood product transfusion were also not available. We recognize these limitations for the exposure and outcome measures as a major drawback in this study.

Neonatal information, post-discharge data, subsequent pregnancy, and long-term medical comorbidity were also not available in the NIS program but these were also important outcome measures for this type of health service outcome research. Accuracy of data was not assessable due to the lack of actual medical record review. Generalizability across different regions or populations was not assessed in this study.

## Conclusion

Despite these limitations, this study raises important considerations in the care of morbidly obese patients proceeding with ART in three areas (preconception period, perinatal care, and intrapartum care) as severely obese patients conceiving through ART are at increased risk for adverse obstetric outcomes and severe maternal morbidity at delivery.

The first area is the preconception period. Counseling about weight loss strategies and referrals to dieticians may be warranted prior to conception. The second area is the perinatal care. Baseline laboratory evaluations, early glucose screening tests, and close monitoring and counseling about maternal weight gain are all key considerations early on in prenatal visits.

The third area is the intrapartum care. Given the increased maternal risks of postpartum hemorrhage and blood transfusion in morbidly obese patients conceived with ART, proper patient referral to the facility with adequate blood product and careful delivery planning for these high-risk pregnancies are recommended.

### Supplementary Information

Below is the link to the electronic supplementary material.

Supplementary file1 (DOCX 27 KB)

## Data Availability

The data that support the findings of this study are openly available in Healthcare Cost and Utilization Project, Agency for Healthcare Research and Quality, at https://www.hcup-us.ahrq.gov/nisoverview.jsp.

## Declarations

### Disclaimer

The funder had no role in the design and conduct of the study; collection, management, analysis, and interpretation of the data; preparation, review, or approval of the manuscript; and decision to submit the manuscript for publication.

### Transparency

The manuscript’s corresponding author (K.M.) affirms that the manuscript is an honest, accurate, and transparent account of the study being reported; that no important aspects of the study have been omitted; and that any discrepancies from the study as planned (and, if relevant, registered) have been explained. The National Inpatient Sample is developed for the Healthcare Cost and Utilization Project that is sponsored by the Agency for Healthcare Research and Quality, and the program is the source of the de-identified data used; race and ethnicity were grouped by the program; and the program has not verified and is not responsible for the statistical validity of the data analysis or the conclusions derived by the study team.

### Abstract presentation

ASRM 2023 Scientific Congress & Expo New Orleans, LA, October 14–18, 2023.
