# Supplementary material for: Pregnancy following assisted reproductive technology in morbidly obese patients: assessment of feto-maternal outcomes
Source: J Assist Reprod Genet. 2024 Feb 21;41(4):903–14. doi: 10.1007/s10815-024-03065-1 (PMC11052942; doi:10.1007/s10815-024-03065-1)
Supplement: Supplementary file 1 — Supplementary file1 (DOCX 27 KB) [file 10815_2024_3065_MOESM1_ESM.docx]

**Supplemental Table S1. ICD-9 codes.**

| Assisted reproductive technology | V23.85 |
| --- | --- |
| Vaginal delivery | 650, 7321, 732.2, 735.9, 736, 372,* 373,* 374,* 375,* 669.50, 669.51, 720, 722.1, 722.9, 723.1, 723.9, 724, 725.1, 725.3, 726, 727.1, 727.9, 728, 729, 669.60, 669.61, 725.1, 725.2, 725.3, 725.4, 726 |
| Cesarean delivery | 649.81, 649.82, 669.70, 669.71, 740, 741, 742, 743, 744, 749.9,  370,* 371* |
| Obesity | 649.1, 278.00, 278.01 |
| Tobacco | 649.0, 305.1, 989.84 |
| Pre-existing hypertension | 642.0, 642.1, 642.2, 642.7 |
| Pre-existing diabetes mellitus | 648.0, 249, 250 |
| Prior cesarean delivery | 654.2 |
| Uterine myoma | 218.0, 218.1, 218.2, 218.9 |
| Multifetal gestation | 651, 662.3, 678.1, 759.4, 989.84, V273, V274, V275, V276, V277, V31, V32, V33, V34, V35, V36, V37, V91 |
| Fetal growth restriction | 656.5, 764.9 |
| Fetal demise | 656.40, 656.41, 656.43, 768.0, 768.1 |
| Breech presentation | 652.20, 652.21, 652.23 |
| Large for gestational age | 656.6 |
| Placenta previa | 641.0, 641.1, 762.0 |
| Placenta abruption | 641.2 |
| Placenta accreta spectrum | PMID: 25957019^†^ |
| Premature rupture of membrane | 658.1 |
| Chorioamnionitis | 658.4, 670.1, 670.2 |
| Gestational hypertension | 642.3 |
| Gestational diabetes mellitus | 648.81, 648.83 |
| Pre-eclampsia | 642.4, 666.7, 642.6, 642.7 |
| Preterm birth | 644.20, 642.21, 765.0, 765.1, 765.21, 765.22, 765.23, 765.24, 765.25, 765.26, 765.27, 765.28 |
| Severe maternal morbidity | ** |
| Hemorrhage | 666.0, 666.1, 666.2, 998.11, E87.00, 285.1 |

* Diagnosis-Related Group (DRG) codes. †Definition was based on the prior study using the panel of ICD-9 codes. **Per the Centers for Disease Control and Prevention (CDC) definition: https://www.cdc.gov/reproductivehealth/maternalinfanthealth/smm/severe-morbidity-ICD.htm
